# A Versatile Method for Uniform Dispersion of Nanocarbons in Metal Matrix Based on Electrostatic Interactions

**DOI:** 10.1007/s40820-015-0061-5

**Published:** 2015-09-18

**Authors:** Zan Li, Genlian Fan, Zhanqiu Tan, Zhiqiang Li, Qiang Guo, Dingbang Xiong, Di Zhang

**Affiliations:** State Key Laboratory of Metal Matrix Composites, Shanghai Jiao Tong University, Shanghai, 200240 People’s Republic of China

**Keywords:** Metal matrix composites, Uniform dispersion, Carbon nanotube, Graphene Electrostatic interactions

## Abstract

Realizing the uniform dispersion of nanocarbons such as carbon nanotube and graphene in metals, is an essential prerequisite to fully exhibit their enhancement effect in mechanical, thermal, and electrical properties of metal matrix composites (MMCs). In this work, we propose an effective method to achieve uniform distribution of nanocarbons in various metal flakes through a slurry-based method. It relies on the electrostatic interactions between the negatively charged nanocarbons and the positively charged metal flakes when mixed in slurry. For case study, flake metal powders (Al, Mg, Ti, Fe, and Cu) were positively charged in aqueous suspension by spontaneous ionization or cationic surface modification. While nanocarbons, given examples as carboxylic multi-walled carbon nanotubes, pristine single-walled carbon nanotube, and carbon nanotube–graphene oxide hybrid were negatively charged by the ionization of oxygen-containing functional groups or anionic surfactant. It was found that through the electrostatic interaction mechanism, all kinds of nanocarbons can be spontaneously and efficiently adsorbed onto the surface of various metal flakes. The development of such a versatile method would provide us great opportunities to fabricate advanced MMCs with appealing properties.

## Introduction

Metal matrix composites (MMCs), defined by dispersing reinforcement materials in metal matrix, have attracted intensive interest in the past decade due to the soaring demand of lighter, stronger, and stiffer structural materials from the field of transportation, aerospace, military, etc. [1]. Although various forms of reinforcements, including particles, fibers, and whiskers, have been applied to MMCs, pursuing a reinforcement material with ideal properties is still desirable to promote the development of high-performance MMCs. Nanocarbons, represented by carbon nanotube (CNT) (1-D) and graphene (2-D), have attracted great interests in recent years because of their excellent properties, both in terms of mechanical and functional fields [2–4]. These fine-scale nanofillers are intrinsically strong while also bringing Orowan strengthening or grain refinement to the metallic matrix, making MMCs stiff, strong, and tough. Besides, they also possess excellent thermal and electrical conductivities, which provide us a chance to achieve MMCs with integrated structural and functional properties [1]. However, the nano-scale feature is also a “double-edged sword” as for reinforcements. While the tremendous specific surface area of those nanocarbons demonstrates great advantages in the field of strengthening, sensors, electrochemical electrodes, and supercapacitors [5, 6], it also results in huge difficulty in dispersing them into metal matrix, because of the strong inter-filler van der Waals interactions and incompatibility between the metal powders and the nanocarbons in terms of the surface properties and morphology [4]. How to incorporate those nanocarbons uniformly into metals is still the main challenge for developing high-performance MMCs.

In the past decade, many methods have been developed to achieve the uniform distribution of nanocarbons in metal matrix and generally can be divided into two categories. One is the bottom-up way, in which nanocarbons such as CNTs and graphene are firstly uniformly dispersed on metal powder surfaces and then they are assembled into composite by consolidation. The in situ growth [7], molecular level mixing [8], layer-by-layer assembling [9], and surface-modified slurry-based method [10] are belonging to this category. Nevertheless, these methods are usually limited to certain MMC systems, or time-consuming or only available for small-sized samples. The other is the top-down manner, in which severe deformation processes such as ball-milling [11], extrusion [12], and friction welding [13] were employed to break the originally agglomerated nanocarbons into the metal matrix. However, the top-down methods have their own drawbacks. The structural integrity of the nanocarbons would be destroyed during severe deformation, leading to the reduced intrinsic properties of reinforcements, as well as serious interface reaction between carbon and certain metal matrix [14]. Although the potential of uniform dispersion of nanocarbons in metal matrix has been demonstrated through the above methods, as far as we know, some nanocarbons, especially for pristine single-walled CNT (SWCNT), have not realized homogenous distribution in even the most studied Al and Mg matrix composite systems. Zhong et al. [15] mixed nano-Al particles with SWCNTs by soaking them in alcohol and stirred ultrasonically in order to realize uniform dispersion of SWCNT in Al matrix; however, SWCNTs bundles were still existed in the final consolidated bulk. George et al. [16] ball milled the mixture powders of Al and SWCNT for 5 min at a relatively lower milling speed to keep the structure of CNT undestroyed; however, the short duration was unable to disperse SWCNT uniformly. Thus, an efficient and non-destructive method for uniformly dispersing of various nanocarbons in metal matrix is utmost needed.

**Fig. 1:**
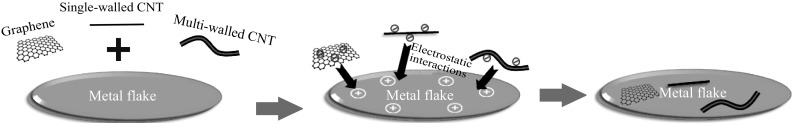
Schematic of the adsorption mechanism of nanocarbons on metal flake powders via electrostatic interactions

Recently, a novel bottom-up fabrication process of flake powder metallurgy was employed to realize a uniform distribution of graphene oxide (GO) in Al matrix based on the adsorption mechanism of electrostatic interaction [17]. This process is very efficient and environmental friendly, and has an ability to fabricate bulk samples with large-scale production potential. This new method relies on the electrostatic interactions between the positively charged Al^3+^ that spontaneously ionized in aqueous suspension and the negatively charged GO containing oxygen functional groups. When they were mixed in an aqueous media, electrostatic interactions would lead to the spontaneous adsorption of GO onto Al flakes. Inspired by the adsorption mechanism, various nanocarbons have a potential to uniformly disperse in metal matrix as long as the surface electron status of the nanocarbons and metal matrixes are modified, which can be illustrated in Fig. 1. Therefore, in the present study, we explored the adsorption mechanism to other nanocarbons (carboxylic multi-walled CNT, pristine SWCNT, and CNT–graphene hybrid)/metal (Al, Mg, Ti, Fe, and Cu) systems. The experimental results demonstrated that all these nanocarbons can be spontaneously and uniformly adsorbed onto the different metal surfaces by the electrostatic interaction adsorption mechanism in an efficient manner. This universal and non-destructive dispersion method would open a door for producing high-performance metal matrix composites reinforced with various nanocarbons.

## Experimental

### Preparation of Nanocarbon/Metal Composite Powders

Generally, three steps were involved during the fabrication process:

#### Preparation of Metal Flakes

Spherical Al, Mg, Ti, Fe, and Cu powders (99.99 % purity, mean particle size 10 μm) were ball milled in a stainless steel mixing jar at a speed of 352 rpm for 3 h in pure ethanol to make metal flakes with thickness of a few hundreds of nanometers. The metal flakes promote the compatibility between the metal powders and the nanocarbons in terms of the surface properties and morphologies [10]. Additionally, the high specific surface area of nanoflakes also greatly promotes the homogeneity and volume fraction of nanocarbons that can be dispersed in metal matrix.

#### Preparation of Nanocarbons Suspension

SWCNT (1–2 nm in diameter, ~1 μm in length, 95 % purity), graphite oxide, and carboxylic multi-walled CNT (MWCNT, 30–50 nm in diameter, ~1 μm in length, carboxylated, 95 % purity) were added to deionized water and then sonicated for 2 h by an ultrasonic homogenizer in ice base to get fully dispersed nanocarbons in aqueous suspensions. During sonication of SWCNT, a kind of anionic surfactant, sodium dodecylbenzene sulfonate (SDBS), was added to the aqueous suspensions. SDBS can be adsorbed on the nanotube surface by the *π*–*π* interaction between the benzene ring and the honeycomb crystal lattice of carbon [18], promoting the SWCNT negatively charged and individually suspended after ultrasonication.

#### Making the Composite Powder by Slurry Mixing

Flake metal powders (20 g) were transferred to a beaker containing 50 ml deionized water. In the slurry, the metal flakes were either spontaneously charged by hydrolysis or surface modified by surfactant to introduce positive charges. To obtain composite powders with a various mass fraction of nanocarbons, the corresponding volume of the nanocarbons’ aqueous suspension was directly poured in and the mixed slurry was stirred at 400 rpm. After the completion of the adsorption process, the slurry in the beaker was filtered, rinsed with ethanol, and finally, vacuum dried at 333 K for 24 h. More details of the adsorption processes can be found in our previous work [17].

### Characterization

Zeta potential measurements of the dispersed nanocarbons and metal flakes in aqueous solutions were conducted by Zeta potential analyzer (Brookhaven ZetaPlus) and ten data for each sample were measured. Scanning electron microscopy (SEM, Quanta 250, FEI) was used to characterize the morphology of the composite powders. Biology Transmission Electron Microscope (TEM, Tecnai G2 spirit Biotwin, FEI) and Raman spectroscopy (Senterra R200-L, Bruker Optics) with an Ar^+^ laser wavelength of 532 nm were applied to analyze the morphology and defects of dispersed and adsorbed SWCNT, respectively.

## Results and Discussion

### Uniform Dispersion of Spontaneously Ionized Nanocarbon in Charged Metal Matrix

**Fig. 2:**
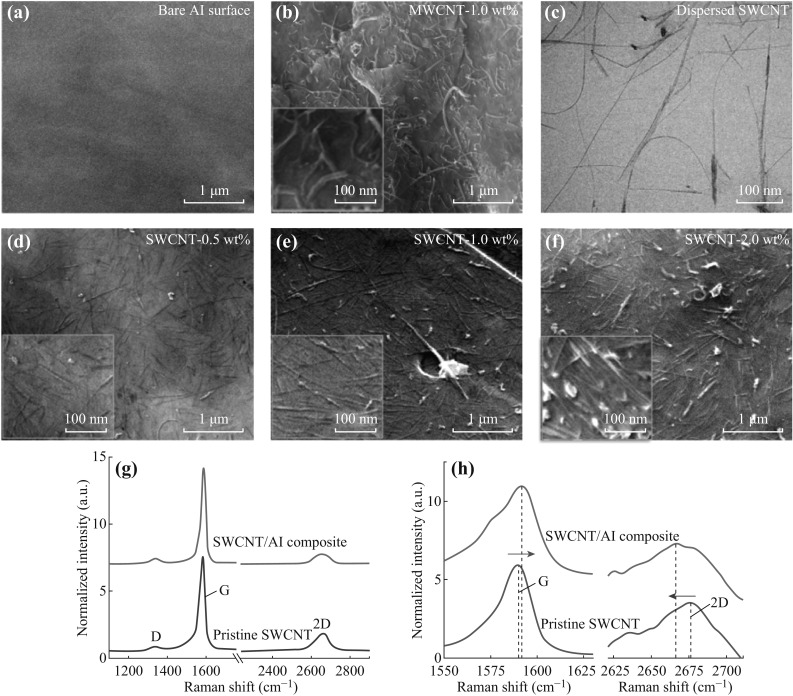
SEM image of Al surface adsorbed with MWCNT: **a** bare Al surface, **b** 1.0 wt% of MWCNT. **c** TEM image of individual dispersed SDBS-modified SWCNT after ultrasonication. SEM images of Al flakes loaded with various contents of SDBS-modified SWCNT: **d** 0.5 wt%, **e** 1.0 wt%, **f** 2.0 wt%. Raman spectrum (**g**) and peaks shift (**h**) of SWCNT/Al composite powders compared with SDBS-modified pristine SWCNT

Metal flakes, which have negative standard reduction potentials (for example Al), have a tendency to ionize and positively charged in aqueous solutions. The carboxyl- like functional groups on carboxylated nanocarbons (for example, carboxylated MWCNT) make it negatively charged in aqueous solution, as evidenced by the negative zeta potential value of sonicated MWCNT (−12.6 ± 1.0 mV). Therefore, when MWCNTs were mixed with metal flakes, which were represented by Al flakes here (Fig. 2a) in aqueous media, they were adsorbed onto Al flakes spontaneously due to the attractive electrostatic interactions, resulting in the uniform distribution on Al flakes as shown in Fig. 2b. The adsorption process can be realized in less than 5 min, and the hydrolysis of metal was not a big problem [17]. This is the easiest case of utilizing the uniform dispersion of nanocarbons in metal matrix by electrostatic interactions.

### Uniform Dispersion of Pristine Non-ionized Nanocarbon in Charged Metal Matrix

For nanocarbons which do not have the ability to naturally ionize, some kind of ionic surfactants was introduced to make them negatively charged. By employing SDBS as surfactant during sonication of SWCNT, the nanotubes were not only individually suspended (Fig. 2c), but also were negatively charged because of the attached dodecylbenzene sulfonate groups (Zeta potential: −9.5 ± 1.3 mV), making the electrostatic adsorption mechanism available for SWCNT/Al system. Figure 2d–f shows the surface of the SWCNT/Al flake composite powders loaded with various mass fractions of SDBS-modified SWCNT. With an increasing mass fraction of SWCNT in composite powders, from 0.5 to 2.0 wt%, the nanotube maintains its uniform distribution on the surface of the Al flakes with few aggregations. Especially, for the modified SWCNT with comparatively large mass fractions, SWCNT was densely packed and well aligned locally as indicated in the inset of Fig. 2f, revealing a transition from randomly to anisotropic distribution of SWCNT with increasing loadings, indicating an entropy-driven alignment of SWCNT during adsorption [19]. The electrostatic adsorption process is a non-destructive dispersion method, and as shown in the Raman spectrum of Fig. 2g, the intensity ratio of D and G peaks (0.085) of SDBS-modified SWCNT/Al composite powders, which arise from lattice disorder at the edges of the *sp* ^2^ clusters and in-plane stretching of the lattice respectively, shows no apparent increment over SDBS-modified pristine SWCNT (0.084). Besides, on average, the G peak is blue shifted by 2 cm^−1^ and the 2D peak (corresponds to high-energy second-order process) is red shifted by 9 cm^−1^ for SWCNT/Al composite powders compared with the surfactant-modified pristine SWCNT (Fig. 2h), suggesting an electron doping of SWCNT by Al matrix, in accordance with the electron transfer-induced reduction effect of GO by Al flake reported in our previous work [17].

**Fig. 3:**
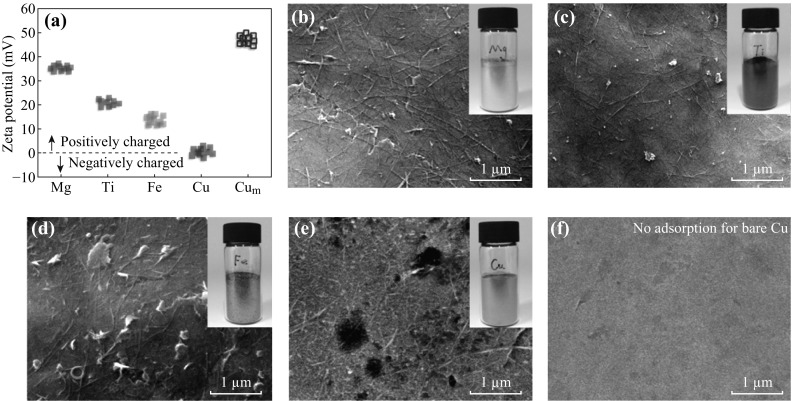
**a** Zeta potential of various metal flakes. Cu_m_ indicates DTAB-modified Cu flakes. **b**–**e** are the SEM images of SDBS-modified SWCNT absorbed composite powders with metal matrix of Mg, Ti, Fe, and Cu, respectively. All flakes are loaded with SWCNT contents of 1.0 wt%. The insets are the optical images of composite flakes. The surface protuberances of modified Cu flakes in (**e**) are DTAB nanoparticles, which can be removed after thermal decomposition. **f** is the SEM image of Cu flake mixed with SDBS-modified SWCNT suspensions for 30 min, which shows that no SWCNT was adsorbed on its surface

The adsorption mechanism of SWCNT in Al matrix was also applicable for other metals which have negative standard reduction potentials. For common metals that are mostly used for structural applications, such as Fe, Ti, and Mg, their ball-milled flakes have the average Zeta potential of 14.3 ± 2.1, 19.8 ± 1.0, and 35.3 ± 1.4 mV, respectively (Fig. 3a), making the spontaneous adsorption of modified pristine SWCNT onto their flake surface possible. Figure 3b–d shows the uniform adsorption of 1.0 wt% of SDBS-modified SWCNT on the surface of Mg, Ti, and Fe flake powders through electrostatic interactions. It is worth mentioning here that due to the large negative standard reduction potential of Mg (−2.37 V, Mg = Mg^2+^ + 2e^−^), it is very easy for Mg flakes to ionize and hydrolyze excessively once in contact with water, and thus, adsorption media containing 60 vol% of water and 40 vol% of ethanol was employed to prevent serious hydrolysis of Mg flakes during the fabrication of SWCNT/Mg composite powders.

### Uniform Dispersion of Pristine Non-ionized Nanocarbon in Non-spontaneously Ionized Metal Matrix

The extreme condition is that both metal flakes and nanocarbons are failed to ionize spontaneously. Different charged surfactants should be applied to modify metal powders and nanocarbons with different charge characters. Cu, with positive standard reduction potential (+0.337 V), has no ability to ionize in aqueous solutions, and SDBS-treated SWCNT can be barely adsorbed on its surface (Fig. 3f). This also indicates that the strength of van der Waals forces between nanocarbons and metal flakes are not strong enough for keeping SWCNT being absorbed on metal flakes. However, after modified by dodecyl trimethyl ammonium bromide (DTAB), a kind of cationic surfactant, the surface of modified Cu flake was positively charged as revealed by the positive Zeta potential value (48.8 ± 2.6 mV) (Fig. 3a). The DTAB solution has a high wetting ability for the Cu flakes, and therefore, DTAB particles with size of ~50 nm can be covered on Cu flakes after mixing Cu powders with DTAB solutions and subsequent filtering, rinsing, and drying of the modified powders. Consequently, through the electrostatic interactions between the positively charged Cu flakes and negatively charged SWCNT, uniform dispersion of SWCNT on Cu flakes can be obtained as revealed in Fig. 3e. The above comparison also reveals that the strength of electrostatic interactions between charged nanocarbons and charged metal flakes is much stronger, as it can keep the nanocarbons attached after mild stirring and filtering process.

All the above results indicate that the electrostatic adsorption mechanism can be expanded to various nanocarbons/metal systems, as long as nanocarbons and metals can be charged by themselves or other surfactants, demonstrating a great universality of the adsorption method.

### Uniform Dispersion of Hybrid Nanocarbons in Metal Matrix

**Fig. 4:**
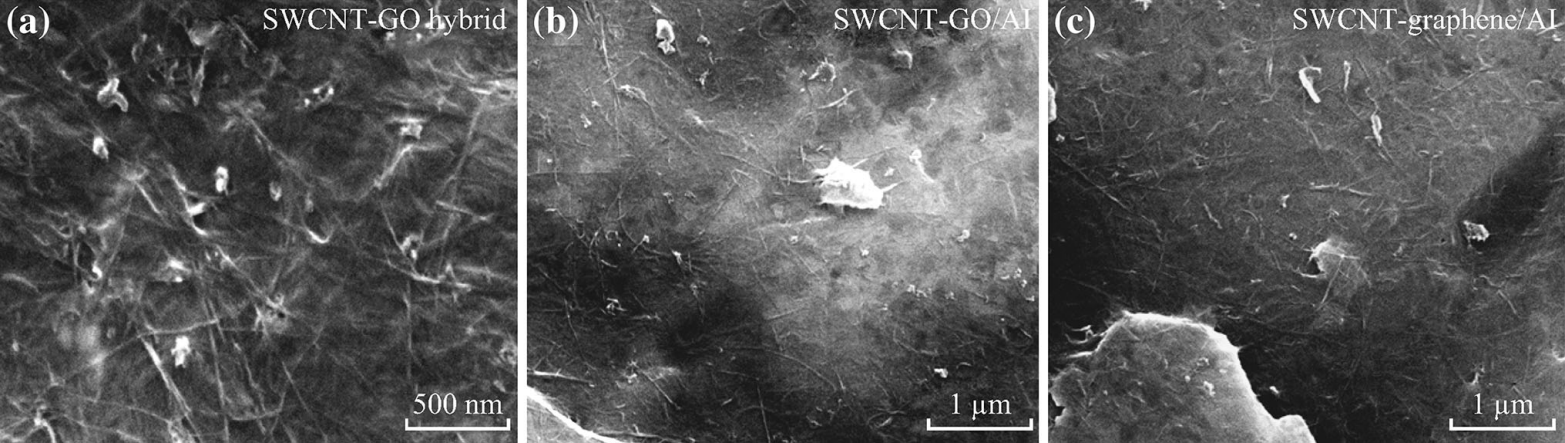
**a** SEM image of the SWCNT–GO hybrid structure. **b**, **c** are SEM images of SWCNT–GO hybrid/Al flake powders after the electrostatic adsorption and SWCNT–graphene/Al flake powders after thermal reduction at 500 °C. The carbon concentration was 1.0 wt%, and the mass ratio of SWCNT to GO is 2:1

Additionally, we will demonstrate that more complex nanocarbon structure, for example, CNT–graphene hybrid, can also be uniformly dispersed in metal matrix through the same adsorption mechanism. The dimensional difference as well as the strong interaction force through π–π interactions promotes spontaneous formation of an interconnected network between SWCNT and GO [20]. As shown in Fig. 4a, SWCNT–GO hybrid was connected together, forming a film-like structure, in which it is difficult to distinguish the ends of each individual SWCNT. The as-formed hybrid was negatively charged (zeta potential: −13.1 ± 0.6 mV), and they can be spontaneously adsorbed onto Al flakes during mixing, as shown in Fig. 4b. The special interconnected hybrid structure has an ability to improve the load transfer efficiency of reinforcements and has revealed a synergistic strengthening effect in polymer and metal matrix [20, 21].

We want to mention that all these nanocarbons can keep their uniform distribution status after removing the functional groups or surfactants during high-temperature heat treatment. Figure 4c shows that SWCNT–graphene/Al composite powders with uniformly dispersed hybrid structure can be kept after a thermal reduction process at 500 °C. It was supposed that the residual functional groups adsorbed on metal flakes after thermal annealing may be responsible for the unchanged uniform distribution of nanocarbons on metal flakes. The flexibility, non-destructive nature, and high efficiency of the dispersion method make the mass production of various nanocarbon-reinforced metal matrix composite powders practicable.

## Conclusions

This work highlights a versatile method of electrostatic adsorption for uniform dispersion of nanocarbons in various metal matrixes. As far as we know, it is the first time that a universal method was applied to achieve different nanocarbons, especially for SWCNT and more complex hybrid structures, uniformly distributed on various metal matrixes, including Al, Mg, Ti, Fe, and Cu. The dispersion process is efficient and non-destructive and has the potential to break the bottom-neck of easy agglomeration of nanocarbons in metal matrix during fabrication. It also provides an inspiration of incorporating other nanofillers uniformly into metal matrix, offering great opportunities to fabricate advanced MMCs with appealing properties.
